# Plasma 25-hydroxyvitamin D deficiency in the peri-operative period is associated with survival outcome in colorectal cancer patients: a meta-analysis

**DOI:** 10.1186/s12893-024-02473-5

**Published:** 2024-06-12

**Authors:** Baojia Zheng, Jianchang Chen, Xiaohua Gong

**Affiliations:** 1 The Fifth Affiliated Hospital of Sun Yat-sen University, Zhuhai, China; 2 The Second People’s Hospital of Xiangzhou District, Zhuhai, China

**Keywords:** Colorectal cancer, 25(OH)D deficiency, Survival outcome, Meta-analysis

## Abstract

**Aim:**

Surgery had a significant impact on 25-hydroxyvitamin D (25-(OH)D) levels. Uncertainty still existed regarding the effects of peri-operative 25(OH)D deficiency on colorectal cancer (CRC) patients’ prognosis. The purpose of the present study was to explore the potential association between the peri-operative 25(OH)D deficiency and the survival outcome of CRC.

**Methods:**

Seven electronic databases [including PubMed, EMBASE, Web of Science, The Cochrane Library, OvidMEDLINE(R), China National Knowledge Infrastructure (CNKI) and Wangfang data] were searched without language limitations. The primary outcomes were overall survival and all-cause mortality. Secondary outcomes were the incidence of 25(OH)D deficiency and risk variables for low 25(OH)D level in the peri-operative period.

**Results:**

14 eligible studies were obtained with 9324 patients for meta-analysis. In the peri-operative period, the pooled incidence of blood 25(OH)D deficiency was 59.61% (95% CI: 45.74–73.48). The incidence of blood 25(OH)D deficiency post-operatively (66.60%) was higher than that pre-operatively (52.65%, 95% CI: 32.94–72.36). Male (RR = 1.09, 95% CI: 1.03–1.16), rectum tumor (RR = 1.23, 95% CI: 1.03–1.47), spring and winter sampling (RR = 1.24, 95% CI: 1.02–1.49) were the risk factors for the 25(OH)D deficiency. The association between the low 25(OH)D post-operatively and short-term overall survival (HR = 0.43, 95% CI: 0.24–0.77) was most prominent, while a low 25(OH)D pre-operatively (HR = 0.47, 95% CI: 0.31–0.70) was more significantly associated with long-term all-cause mortality than that after surgery.

**Conclusion:**

Peri-operative 25(OH)D impacted the CRC patients’ prognosis. Due to possible confounding effects of systemic inflammatory response (SIR), simultaneous measurement of vitamin D and SIR is essential for colorectal survival.

**Supplementary Information:**

The online version contains supplementary material available at 10.1186/s12893-024-02473-5.

## Introduction

Colorectal cancer (CRC) was currently the fourth most common cancer worldwide, and its incidence was still on the rise [1]. In the United States, CRC was the second most common cause of cancer death [2]. The mainstay of treatment for colorectal cancer was still surgery [3]. Despite advances in treatment over the past three decades, the mortality rate of CRC remained high mostly due to recurrence and distant organ metastases [4]. It was widely reported that tumor characteristics were not the only factor influencing prognosis, while host-related factors in the peri-operative period were greatly contributing to the poor survival outcomes in patients with CRC [5]. Up to now, it had been discovered that peri-operative status of some host factors, such as anima [6], sarcopenia [7] and immune status [8] played substantial roles in tumor growth, which were closely associated with prognosis. Thus, exploring other possible peri-operative host-factors associated to prognosis and taking early intervention possibly benefited the prognosis and survival of CRC.

Vitamin D was a steroid hormone known to affect calcium homeostasis and skeletal physiology [9]. Due to the strong homeostatic regulation of the formation and levels of 1,25(OH)2D and the short half-life, studies frequently used circulating 25-hydroxyvitamin D (25(OH)D) to determine vitamin D status [10]. Vitamin D status in human disorders had drawn increasing attention, which was recognized as a kind of crucial hormone for preserving the regular operation of many organs or bodily systems [11]. A recent meta-analysis confirmed that higher vitamin D intake and serum 25(OH)D levels were associated with lower cancer risk and cancer-related mortality [12]. Numerous research and meta-analyses had revealed that adequate vitamin D consumption reduced the onset of CRC [13, 14]. Besides, vitamin D might act as a predictive factor for the survival outcome in CRC [15, 16]. The level of 25(OH)D concentration significantly fluctuated during surgery [17]. However, peri-operative vitamin D levels still had ambiguous findings on prognosis in patients with CRC [18, 19].

Therefore, the meta-analysis was conducted to further identify the impact of the peri-operative vitamin D levels on short-term and long-term survival outcome in CRC. Simultaneously, the incidence and risk factors of peri-operative 25(OH)D deficiency were further evaluated to assist in the clinical identification of evidence for intervention.

## Materials and methods

### Search strategy

The present review adhered to Preferred Reporting Items For Systematic Reviews and Meta-Analyses(PRISMA) statement [20]. In the last week of April 2023, a search was conducted in seven electronic databases, including PubMed, EMBASE, Web of Science, The Cochrane Library, OvidMEDLINE(R), China National Knowledge Infrastructure (CNKI) and Wangfang data, without language limitations. The following search terms (MeSH) were used in “All fields” to identify relevant published articles:

1. “CRC” OR “colorectal cancer” OR “colorectal tumor” OR “colorectal neoplasms” OR “colon cancer” OR “rectal cancer” OR “rectal neoplasm” OR “colonic neoplasm” OR “colon” OR “rectum”.
2. “25(OH)D” OR “25-hydroxyvitamin D” OR “vitamin D”.
3. “surgery” OR “operation”.
4. “prognosis” OR “mortality” OR “survival” OR “outcomes”.
5. 1 AND 2 AND 3 AND 4.

### Study selection

**Fig. 1:**
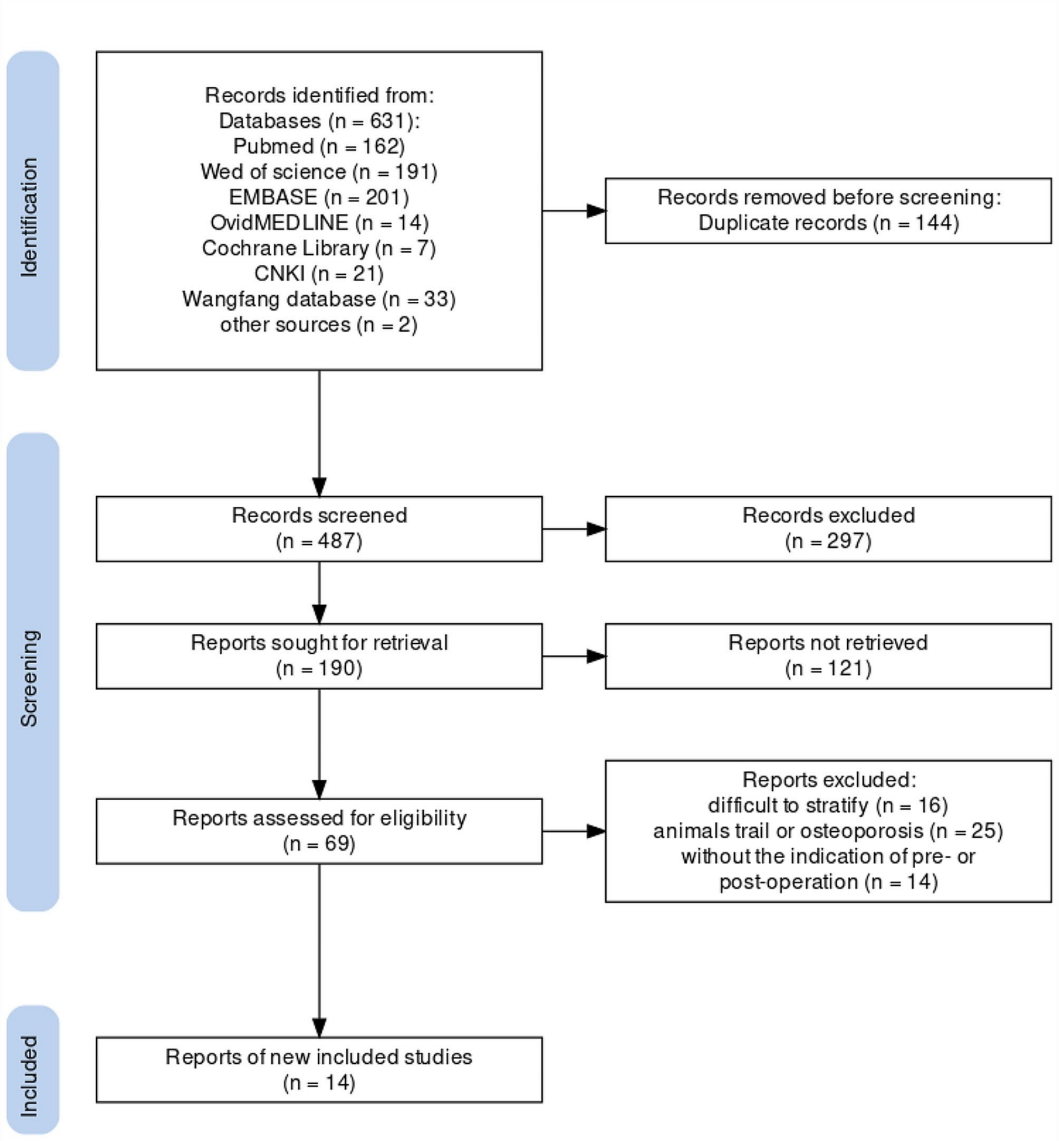
Flow diagram for study selection

The published studies were retrieved with no restrictions of language. The included studies were required to meet the following eligible criteria: (a)prospective or retroprospective cohort studies; (b) measurement of circulating 25-OHD in patients with colorectal cancer; (c) description of the outcome in colorectal cancer; and (d) reporting HRs and corresponding 95%CIs according to different categories of blood 25(OH)D levels to conduct a meta-analysis. Reports published as review articles, protocol, editorials, guidance and consensus were excluded. All titles were independently screened by two authors in accordance with the search strategy mentioned above (B.Z and J.H). A total of 631 records were acquired from the 7 electronic databases. After removing duplicate records, two authors independently performed an abstract review of potentially relevant records to assess their eligibility based on the inclusion criteria. Differences were resolved through discussion until a consensus was reached. Then, a full text search of the remaining 69 records was conducted, and 55 records were eliminated due to animals trail, or without the indication of the pre- or post-operation, or difficulty in stratifying **(**Fig. 1**)**. The Newcastle Ottawa Quality scale (NOS) for assessing the quality in meta-analyses was used to evaluated the quality of the included studies [21]. For NOS, a score of at least 6 out of 9 indicated high quality.

### Data extraction

The data from eligible studies were extracted including the following information: first author’s name, publication year, country, study design, time for blood drawing, sample size, gender ratio(male/female), median age, follow-up duration, CRC stage, death, cut-off value of 25(OH)D deficiency, tertiles for outcome analysis, HR and 95%CI for overall survival and all-cause mortality in the studies.

### Statistical analysis

The primary outcomes were the impact of the levels of peri-operative 25(OH)D on overall survival and all-cause mortality. Secondary outcomes were the incidence and risk factors of 25(OH)D deficiency in the peri-operative period. Pooled proportions were calculated for incidence of 25(OH)D deficiency by conducting single-arm meta-analysis, meanwhile subgroup meta-analyses were individually performed for all variables based on pre-operative point and post-operative point, short-term and long-term survival outcome. The binomial distribution was utilized to calculate risk ratio (RR) and 95% confidence interval (CI) for risk factors. The extracted HRs and 95% CIs were used to calculate the pooled HR estimates. A random-effects model was used for all meta-analysis because the significant heterogeneity was assumed among the included studies [22]. A forest plot was used to illustrate the distribution of the outcome and effect size obtained from each included study. Egger’s test was used to detect the potential publication bias. All statistical analyses were conducted using STATA 16.0 (STATA Corp, College Station, TX).

## Results

### Characteristics of the included studies

The flow diagram of the search and study selection process was shown in Fig. 1. Characteristics of the included studies [17, 18, 23–34] were summarized in Table 1. A total of 14 eligible studies with 9324 patients (male: 5541, female: 3783) were included. The numbers of patients in the included studies ranged from 56 to 1848 cases. The overall average age (± standard error [SE]) of the all patients was 64.09 ± 2.11 years. These studies were published between 2014 and 2021, of which twelve were prospective studies and two were retrospective studies. Of these studies, five were from Asia (three from China, one from Japan and one from Singapore) and nine were from outside Asia (two from the UK, two from Turkey, two from Netherlands, one from Finland, one from Ireland, and one from Norway). The cut-off values of 25(OD)D deficiency and the tertiles for outcome analysis in different studies were shown for pooled extraction. Overall survival and all-cause mortality [high level vs. low level of 25(OH)D] in the included studies were also revealed in Table 1. In addition, NOS scores represented the quality of all included studies based on the NOS checklist, of which 11 studies were at least 6, indicating high quality of adherence for most studies.

**Table 1:** Characteristics of the included studies

Author (year, country)	Study design	Time for blood drawing (median time)	No. of patients	Gender ratio (male/female)	Median age	Follow-up (months)	CRC stage	Death	Cut-off values of 25(OH)D deficiency	Tertiles for outcome analysis	Overall survial HR(95%CI)	All-cause mortality HR(95%CI)	NOS scores
Markotic et al., (2019,Ireland) [23]	prospective	pre-operative cohort: 1 day before surgery; post-operative cohort: 8 days after surgery (median time)	pre-operative cohort:286; post-operative cohort: 229	pre-operative cohort: 184/102; post-operative cohort: 137/92	pre-operative cohort: 65.9 ± 11.6; post-operative cohort: 65.7 ± 11.7	70.8	I-III	pre-operative cohort:118; post-operative cohort: 113	≤ 50nmol/l	≤ 50nmol/l, >50nmol/l	pre-operative cohort: 1.13(0.77–1.65); post-operative cohort: 0.53(0.33–0.84)	/	7
Wesselink et al., (2021, Netherlands ) [24]	prospective	at diagnosis	679	440/239	67	42	I-III	68	< 50nmol/L	< 50 nmol/L; ≥ 50 nmol/L	/	0.39(0.21–0.73)	7
Väyrynen et al., (2016,Finland) [17]	prospective	pre-operative	117	58/59	/	60	I-IV	/	/	≤ 50nmol/l, >50nmol/l	0.80 (0.43–1.49)	/	7
Ng et al., (2018,Singapore) [18]	prospective	17 days after surgery	56	34/22	62	22	II- III	/	< 20ng/ml	<21ng/ml, 21-30ng/ml, >30ng/ml	0.30(0.04–2.44)	/	5
Vaughan-Shaw et al., (2019,UK) [25]	prospective	cohort 1: postoperative ; cohort 2: peri -operative	cohort 1 :1687; cohort 2 :1848	cohort 1 :970/717; cohort 2 :1024/824	/	cohort 1 :159.6; cohort 2 :43.2	I-III	cohort 1 :709; cohort 2 :211	/	cohort 1 : >18.1,18.1–33.1, >33.1nmol/l;; cohort 2 : <38.0,38.0-57.9, >57.9nmol/L	cohort 1:0.68(0.50–0.85)	cohort 1 : 0.65(0.51–0.81); cohort 2 : 0.63(0.44–0.89)	8
Zgaga et al., (2014,UK) [26]	prospective	105 days after surgery	1598	916/682	/	106.8	I-III	531	<10ng/ml	<7.25ng/ml, 7.25—13.25ng/ml, >13.25ng/ml	/	0.70(0.55–0.89)	9
Yang et al., (2017,China) [27]	prospective	within seven days before operation	206	131/75	63	45	I-III	87	<6.2ng/ml	<6.2ng/ml, 6.2-29.9ng/ml, >29.9ng/ml	0.442(0.238–0.819)	/	6
Akinci et al., (2014, Turkey) [28]	retrospective	post-operative: at the remission period	113	79/34	/	48	I-IV	/	< 30ng/ml	/	/	/	5
Mezawa et al., (2010, Tokyo) [29]	prospective	the peri-operative period	257	165/92	65	32.4	I-IV	39	< 30ng/ml	3-7ng/mL;8–10 ng/mL; 11–15 ng/mL; 16–36 ng/mL	0.91(084-0.99)	/	7
Abrahamsson et al., (2021,Norway) [30]	prospective	2–3 days prior to histological confirmation	129	83/46	65	39	I-IV	35	< 50nmol/L	/	/	/	6
Balci et al., (2021, Turkey) [31]	prospective	in a week to the scheduled operation	104	64/40	62.71	/	I-IV	/	< 20ng/ml	/	/	/	5
Bao et al., (2020, China) [32]	retroprospective	before surgery	the primary cohort:523; the validation cohort:205	the primary cohort:304/219; the validation cohort:120/85	61	the primary cohort: 64.7; the validation cohort:53	II-III	81	< 75 nmol/L	<47.5nmol/L, ≥ 47.5nmol/L	0.564 (0.352–0.904)	/	7
Wesselink et al., (2020, Netherlands ) [33]	prospective	at diagnosis	1169	751/418	67	/	I-III	/	< 50nmol/L	< 50 nmol/L; ≥ 50 nmol/L	/	0.53(0.31–0.89)	8
Xingyu et al., (2020,China) [34]	prospective	before surgery	118	81/37	/	/	/	/	< 20ng/ml	/	/	/	5
Total			9324	5541/3783	64.09 ± 2.11								

### Incidence and risk factors of blood 25(OH)D deficiency in the peri-operative period

**Fig. 2:**
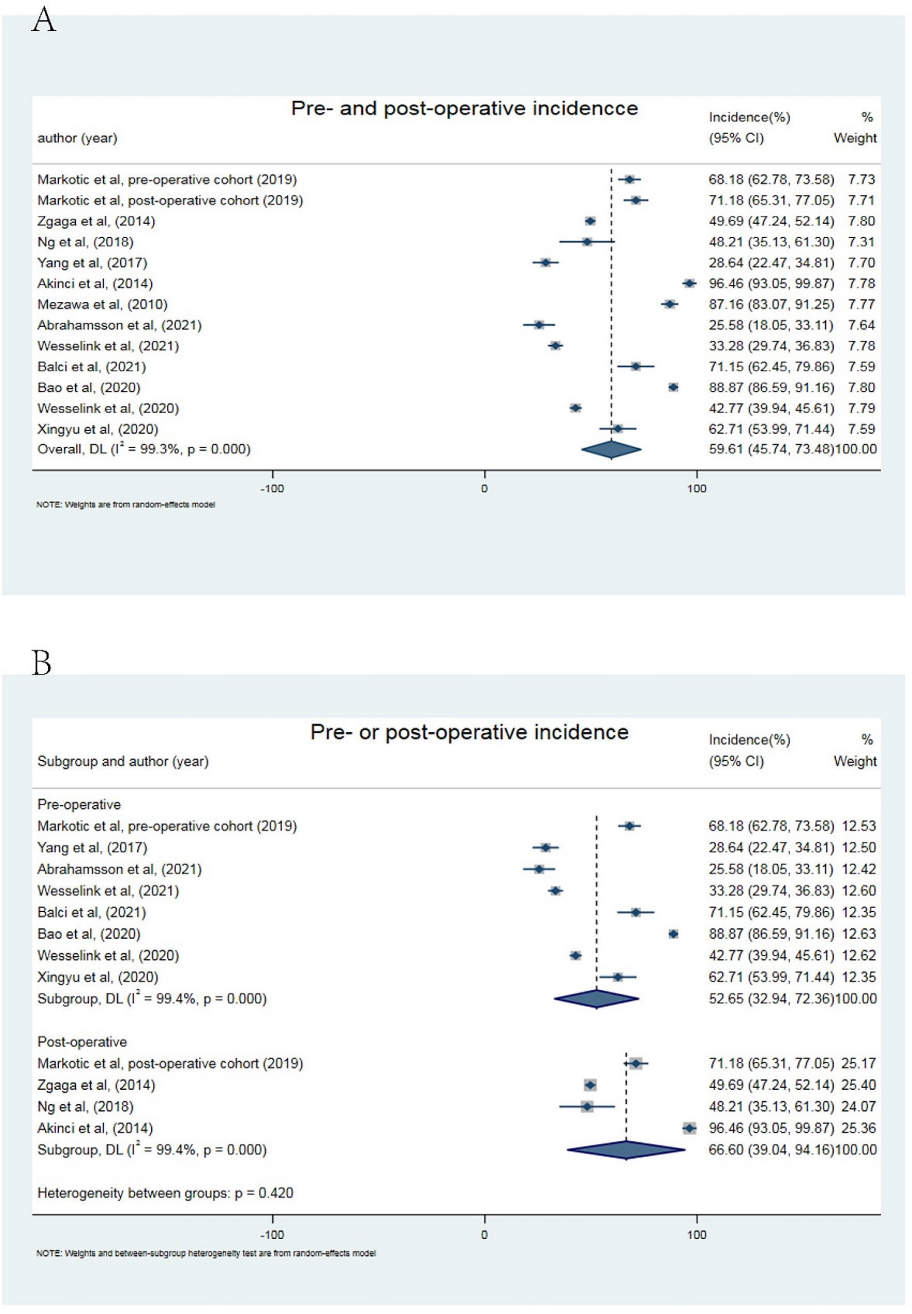
Estimated incidence of peri-operative blood 25(OH)D deficiency in colorectal cancer. **(A)** Single-arm meta-analysis of the incidence of blood 25(OH)D deficiency in pre- and post-operative period in enrolled cases. **(B)** Subgroup analysis of the incidence of blood 25(OH)D deficiency in pre- or post-operative period in enrolled cases by using single-arm meta-analysis

**Fig. 3:**
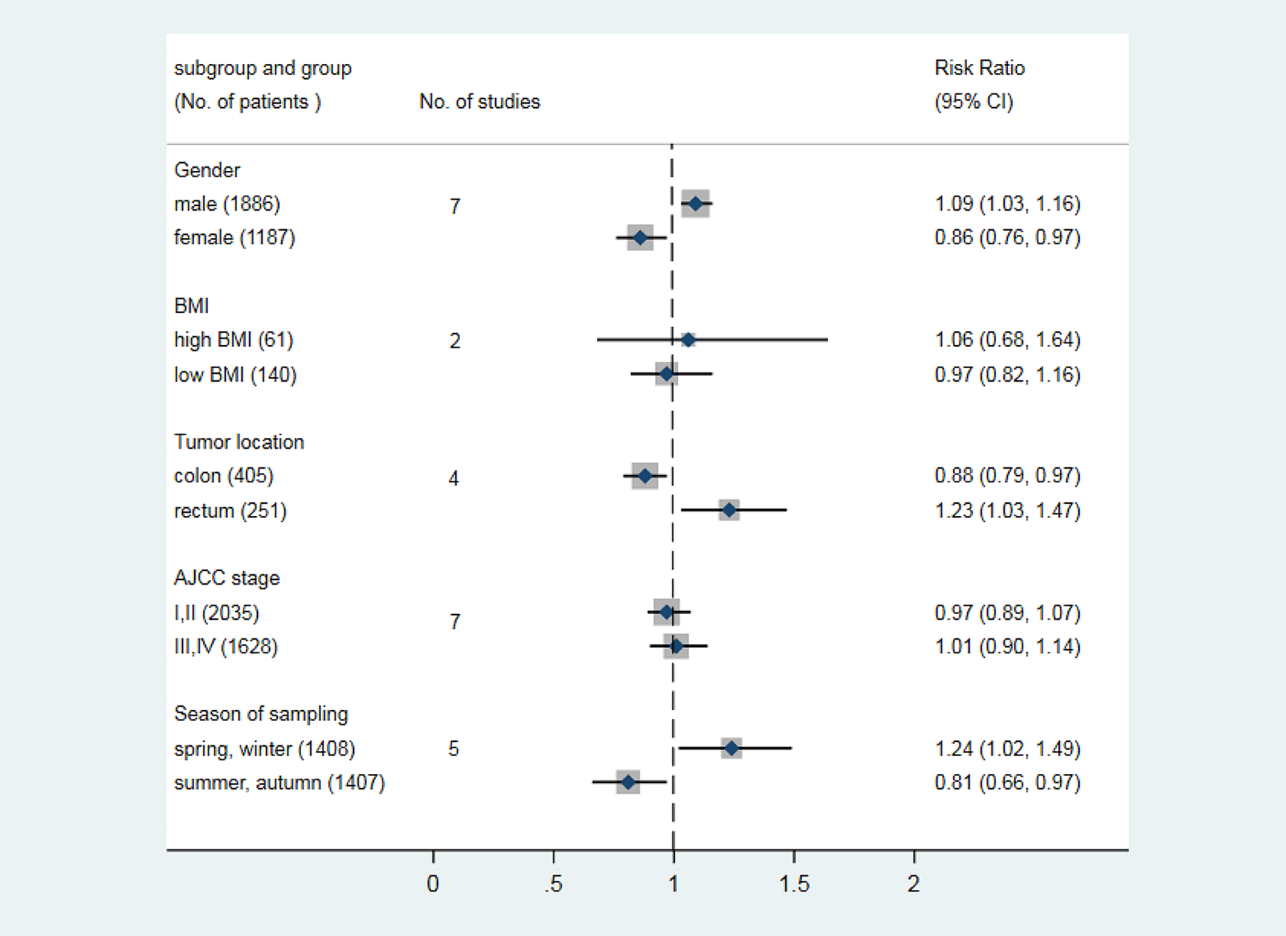
Subgroup analysis of risk factor for peri-operative blood 25(OH)D deficiency in patients with colorectal cancer

The cut-off values of blood 25(OH)D deficiency from the included studies were presented in Table 1. As shown in Fig. 2A, the incidence of peri-operative blood 25(OH)D deficiency was ranging from 25.58 to 96.46%, estimated of 59.61% (95% CI, 45.74–73.48) in the peri-operative period. Furthermore, the incidence of blood 25(OH)D deficiency post-operatively was higher than that pre-operatively, reaching for 66.60% (95% CI: 39.09–94.16; Fig. 2B) with significant heterogeneity. Male, rectum tumor, spring and winter sampling were the risk factors for peri-operatively blood 25(OH)D deficiency, with a pooled RR of 1.09 (95% CI: 1.03–1.16) in male; 1.23 (95% CI: 1.03–1.47) in rectum tumor and 1.24 (95% CI: 1.02–1.49) in spring and winter sampling, more detail shown in Fig. 3.

### Meta-analysis for overall survival

**Fig. 4:**
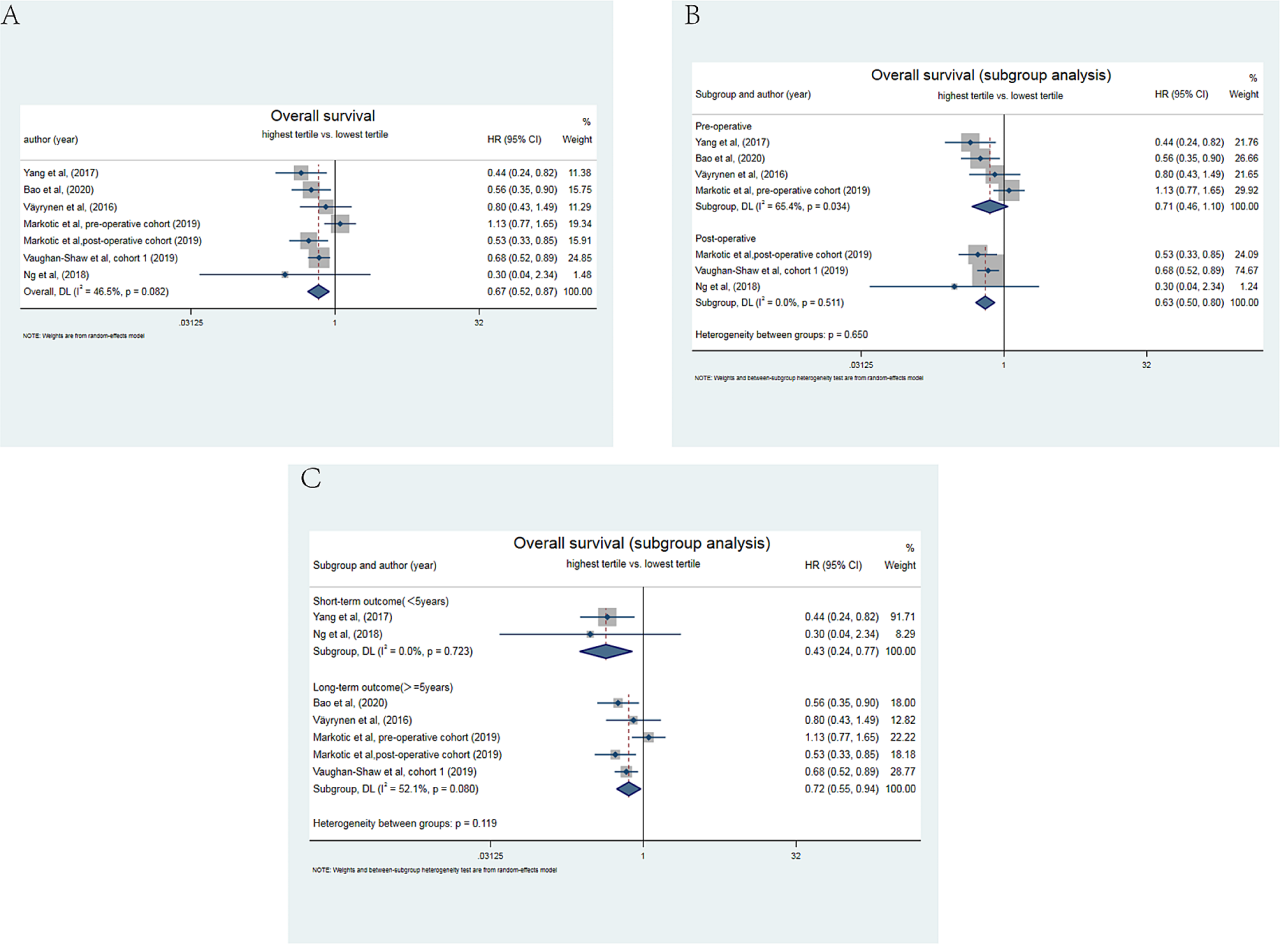
Meta-analysis of the effect of peri-operative blood 25(OH)D level on overall survival. **(A)** Forest plot for the association of highest vs. lowest 25(OH)D levels in pre- and post-operative period with overall survival in patients with colorectal cancer. **(B)** Subgroup analysis of forest plot for the association of highest vs. lowest 25(OH)D levels in pre- or post-operative period with overall survival in patients with colorectal cancer. **(C)** Subgroup analysis of forest plot for the association of highest vs. lowest 25(OH)D levels in short term(< 5 years) or long term (≥ 5 years) with overall survival in patients with colorectal cancer

The associations between low peri-operatively blood 25(OH)D and overall survival(OS) were reported in seven cohorts with 3104 cases (Fig. 4). Based on a random-effects model (*I*^*2*^ = 46.5%), the combined result showed that low peri-operative blood 25(OH)D was significantly associated with poor OS (Fig. 4A, HR = 0.67, 95% CI = 0.52–0.87) when the highest tertiles compared with the lowest tertiles. The subgroup analysis between the pre- or post-operative time-point were conducted, and found that low post-operatively blood 25(OH)D (HR = 0.63, 95% CI: 0.50–0.80; *I*^*2*^ = 0%) was more likely to contribute to pooer OS than low pre-operative level (HR = 0.71, 95% CI: 0.46–1.10) as shown in Fig. 4B. Furthermore, in terms of the impact on short-term survival outcome (< 5 years) and long-term survival outcome (≥ 5 years), the pooled results showed that low peri-operative level of blood 25(OH)D affected both short-term and long-term survival outcome. And the HR for short-term survival outcome was 0.43 (95%CI:0.24–0.77, Fig. 4C) without heterogeneity, while the HR for long-term survival outcome was 0.72 (95%CI:0.55–0.94) with the *I*^*2*^ of 52.1%. The funnel plot showed in Supplemental Fig. 1A and *p* value of Egger’s test was 0.055, indicating no potential publication bias.

### Meta-analysis for all-cause mortality

**Fig. 5:**
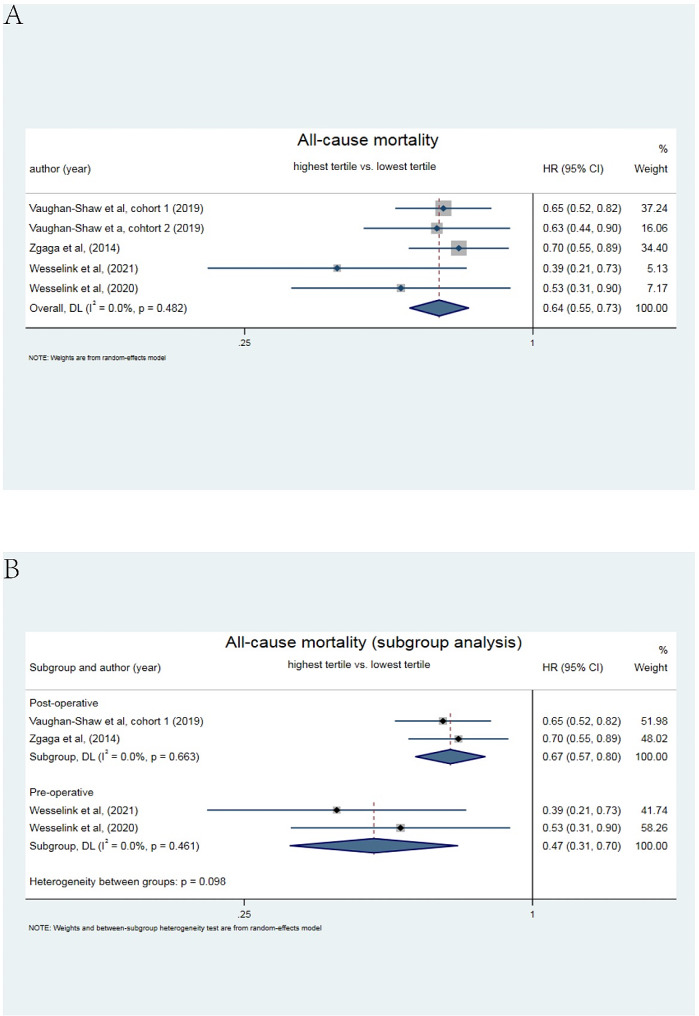
Meta-analysis of the effect of peri-operative blood 25(OH)D level on all-cause mortality. **(A)** Forest plot for the association of highest vs. lowest 25(OH)D levels in pre- and post-operative period with all-cause mortality in patients with colorectal cancer. **(B)** Subgroup analysis of forest plot for the association of highest vs. lowest 25(OH)D levels in pre- or post-operative period with all-cause mortality in patients with colorectal cancer

The effects of peri-operative blood 25(OH)D status on all-cause mortality in CRC patients were also assessed in the present study. Five cohorts with long-term survival outcome (larger than 5 years) comprising 6981 patients reported the prognostic value of blood 25(OH)D in the peri-operative period for all-cause mortality (Fig. 5A). The results suggested that lower level of peri-operative blood 25(OH)D was strongly associated with higher all-cause mortality (HR = 0.64, 95% CI: 0.55–0.73) without heterogeneity (*I*^2^ = 0.0%). As shown in Fig. 5B, the subgroup analysis showed that lower blood 25(OH)D status pre-operatively (HR = 0.47, 95% CI:0.31–0.70) was inclined to have higher all-cause mortality than lower blood 25(OH)D post-operatively (HR = 0.67, 95% CI: 0.57–0.80) with an *I*^*2*^ value of 0%. However, the funnel plot was observed and Egger’s test (*p* = 0.03; Supplemental Fig. 1B) implied a potential publication bias.

## Discussion

Adequate data suggested getting enough vitamin D could lower the risk of formation and incidence in CRC, highlighting the important role of vitamin D in CRC prevention [35]. Furthermore, the vitro studies also found that vitamin D evidently regulated cell proliferation and apoptosis, and promoted cellular differentiation to prevent the development of cancer [36]. However, the impact of vitamin D levels in peri-operative period on CRC patients’ prognosis was still inconsistent. It was challenging to provide precise time of intervention due to failure in specifying the exact time of blood collection, resulting from ignoring effects of peri-operative time of blood 25(0 H)D on CRC`s treatment and outcomes, even though a few prior articles suggested the level of circulating vitamin D was associated with the prognosis in colorectal patients [37, 38]. Furthermore, vitamin D levels were fluctuating greatly and rapidly during surgery, which might lasting for three months or longer before the concentration recovers to “normal” levels [39]. So an integrated meta-analysis was conducted to identify the role of peri-operative blood 25(0 H)D in survival outcome in CRC`s patients. It was found that the incidence of peri-operative vitamin D deficiency was pretty high and did have a significantly negative impact on survival outcome.

14 studies were included comprising 9324 CRC’s patients in this systematic review. The findings revealed that the incidence of peri-operatively blood 25(OH)D deficiency had significantly over 50%, despite some substantial variability due to regional variations in vitamin D status [40]. Additionally, it was demonstrated by the estimated data from subgroup meta-analysis that the incidence of blood 25(OH)D deficit in post-operative period was higher than that in pre-operative period. As Vaughan-Shaw et al. previously reported, the authors found that blood 25(OH)D levels were lower in the twelve months following surgery, then recovering after twenty-four months [25]. The incidence of 25(OH)D deficiency after surgery was higher than that before surgery as shown in our findings, which was more likely to be caused by plasma loss during surgery or peri-operative anxiety, stress, fasting and other conditions [41]. Therefore, the impact of the deficiency in peri-operative period on tumor metastases and deterioration did warrant more investigation, even though 25(OH)D was more reported to be related to the onset of colorectal cancer [41, 42].

In the current study, the potential risk factors for 25(OH)D deficiency peri-operatively were also analyzed and revealed that male, rectum tumor, spring, and winter sampling were the significant risk variables. For sex deference, some literature evidenced the importance of some gender-specific determinants of circulating 25(OH)D as factors to be considered rather than straight differences in vitamin D basal levels between males and females [43]. As an example, higher incidence of colorectal carcinomas in men had been linked to male sex hormones compared with women [44]. Additionally, smoking [45] and low physical activity [46] seemed predictors of vitamin D deficiency in men, which exerted a continuous impact on vitamin D status in the peri-operative period. On the other hand, meta-analysis showed that rectum cancer had higher risk occurring peri-operative 25(0 H)D deficiency when compared with colon cancer, which might be related to rectal mucosal gene expression. It was found that normal rectal mucosal gene expression signature could induce higher 25-OHD level [47], while partial rectal resection and mucosal damage resulting from surgery probably lead to reduced plasma vitamin D level. The reduction in blood 25(0 H)D samples taken in spring and winter correlated with the lack of seasonal sunlight exposure, since adequate sunlight exposure was one of the main sources of vitamin D supplementation [48]. However, BMI and ACJJ stage were not the significant risk factors for low blood 25(0 H)D levels in this study. However, it is worth noting that in both inflammatory bowel disease (IBD) and Crohn’s disease (CD), there was a negative association between BMI and vitamin D serum levels [49]. A previous systematic review showed significant but weak correlation between BMI and 25-OHD in adults [50], However, another’s report revealed that BMI was not an independent predictor of low 25-OHD levels [17], so whether BMI was a risk factor for low plasma 25-OHD levels during CRC`s treatment remains controversial.

Low circulating 25(OH)D concentration throughout peri-operative period was greatly associated with overall survival and all-cause mortality, indicating that the vitamin D fluctuation and deficiency during surgery could result in adverse prognosis. As for overall survival, the subgroup analysis was conducted and revealed that association between the post-operative 25(OH)D and overall survival was more prominent than that before surgery. To our best knowledge, this was the first time to identify the role of the post-operative blood 25(OH)D level on overall survival in CRC by systematic review, especially in short-term survival(< 5 years), which facilitated to provide a time point for target clinical intervention. Animal researches showed that sufficient vitamin D could hinder the progression of CRC through multiple pathways, including Wnt/β-catenin, apoptosis, and inflammation [51]. Furthermore, vitamin D could enhance survival of CRC`s patients through increasing immune modulation [52], attenuation of fatigue symptoms [53], and a decrease of mood disorders or depression [54]. As mentioned above, post-operative vitamin D deficiency was highly prevalent and sustained for a long time, which could lead to dysregulation of anticancer pathways, contributing to the deterioration of CRC. On the contrary, patients with a high vitamin D level after surgery through supplementation presented better disease-free survival and overall survival than patients with low vitamin D level [55], which fostered the evidence that the importance of follow-up monitoring and supplementation of vitamin D level after surgery for CRC patients.

The role of peri-operative 25(OH)D level in all-cause mortality was also evaluated in this study. Although CRC-specific mortality might have more reference value, it was difficult to meta because the small number of articles describing CRC-specific mortality associated with the peri-operative 25(OH)D level. All articles for analyzing all-cause mortality included followed-up lasting for over 5 years, and the finding revealed that low peri-operative blood 25(OH)D level also resulted in the elevated all-cause mortality. Interestingly, the impact of low 25(OH)D pre-operatively was more pronounced than post-operatively on all-cause mortality. Inflammation was possibly one of the potential underlying processes explaining the link between vitamin D and all-cause mortality [56]. Cancer progression was significantly influenced by inflammation [57] and vitamin D’s active form (1,25-dihydroxycholecalciferol) has anti-inflammatory characteristics [58]. Inflammation might contribute to the development of comorbid conditions like cardiovascular disease [59], which was experienced by many cancer survivors [60]. Not to be overlooked, the systemic inflammatory response (SIR) possibly had an important confounding effect in the correlation between vitamin D and cancer survival outcomes, with some scholars suggesting that low plasma 25(OH) concentrations were a consequence of persistent SlR, as there was a recognized association between the presence of SIR and poor prognosis in cancer patients [61]. There was consistent evidence that plasma levels of the most common micronutrients, such as vitamin D, decreased progressively in mild, severe to profound inflammation [62]. Clearly, most plasma micronutrients were part of the systemic inflammatory response. It was recommended that measurements of plasma micronutrient concentrations should be performed in conjunction with measurements of inflammatory responses in patients with acute or chronic disease. However, few of the included studies analyzed the association between SIR markers and vitamin D, resulting in a lack of meta-analysis to verify.

This study had some limitations. Firstly, the number of studies included was still small. Although seven large database were searched and the references of included studies were checked, we cannot exclude having missed a relevant study. Second, there was significant heterogeneity in the combined results of the incidence of blood 25(OH)D deficiency peri-operatively due to regional and population variations. There were variations in units and cut-off values because of different trials and cohorts, but all our meta-analyses were comparing highest level and lowest level. And overall results were remarkably consistent across studies without heterogeneity. For the exact time of blood drawing in our included studies, the pre-operative time was at diagnosis or within a week before surgery, while the post-operative time was mostly within a week or a month after surgery and only one study was three months after surgery, but the results of meta-analysis were generally consistent after sensitivity analysis.

## Conclusion

There was a high peri-operative incidence of circulating 25(OH)D deficiency in CRC`s patients. Both the pre-operative and post-operative vitamin D level was greatly associated with the survival outcome in CRC patients. Due to the possible confounding effect of SIR in the correlation between vitamin D levels and colorectal survival, simultaneous measurement of vitamin D and SIR in the peri-operative period are essential for improving CRC`s survival outcome.

### Electronic supplementary material

Below is the link to the electronic supplementary material.

Supplementary Material 1

## Data Availability

All the data are available without restriction. Researchers can obtain data by contacting the corresponding author.

## References

[1] Sung H, Ferlay J, Siegel RL, Laversanne M (2021). Global Cancer statistics 2020: GLOBOCAN estimates of incidence and Mortality Worldwide for 36 cancers in 185 countries. CA Cancer J Clin.

[2] Siegel RL, Miller KD, Goding Sauer A (2020). Colorectal cancer statistics, 2020. CA Cancer J Clin.

[3] Pramateftakis MG (2010). Optimizing colonic cancer surgery: high ligation and complete mesocolic excision during right hemicolectomy. Tech Coloproctol.

[4] Xu RH, Muro K, Morita S (2018). Modified XELIRI (capecitabine plus irinotecan) versus FOLFIRI (leucovorin, fluorouracil, and irinotecan), both either with or without bevacizumab, as second-line therapy for metastatic colorectal cancer (AXEPT): a multicentre, open-label, randomised, non-inferiority, phase 3 trial. Lancet Oncol.

[5] Miller KD, Nogueira L, Devasia T (2022). Cancer treatment and survivorship statistics, 2022. CA Cancer J Clin.

[6] Kwon YH, Lim HK, Kim MJ (2020). Impacts of anemia and transfusion on oncologic outcomes in patients undergoing surgery for colorectal cancer. Int J Colorectal Dis.

[7] Xie H, Wei L, Liu M, Yuan G, Tang S, Gan J (2021). Preoperative computed tomography-assessed Sarcopenia as a predictor of complications and long-term prognosis in patients with colorectal cancer: a systematic review and meta-analysis. Langenbecks Arch Surg.

[8] Chen JH, Zhai ET, Yuan YJ (2017). Systemic immune-inflammation index for predicting prognosis of colorectal cancer. World J Gastroenterol.

[9] Zmijewski MA (2019). Vitamin D and Human Health. Int J Mol Sci.

[10] Holick MF (2007). Vitamin D deficiency. N Engl J Med.

[11] Urashima M, Ohdaira H, Akutsu T (2019). Effect of vitamin D supplementation on Relapse-Free Survival among patients with Digestive Tract cancers: the AMATERASU Randomized Clinical Trial. JAMA.

[12] Arayici ME, Basbinar Y, Ellidokuz H, Vitamin D, Intake (2023). Serum 25-Hydroxyvitamin-D (25(OH)D) levels, and Cancer Risk: a Comprehensive Meta-Meta-Analysis including Meta-analyses of randomized controlled trials and Observational Epidemiological studies. Nutrients.

[13] Xu Y, Qian M, Hong J (2021). The effect of vitamin D on the occurrence and development of colorectal cancer: a systematic review and meta-analysis. Int J Colorectal Dis.

[14] Boughanem H, Canudas S, Hernandez-Alonso P (2021). Vitamin D intake and the risk of Colorectal Cancer: an updated Meta-analysis and systematic review of case-control and prospective cohort studies. Cancers (Basel).

[15] Maalmi H, Walter V, Jansen L (2017). Relationship of very low serum 25-hydroxyvitamin D3 levels with long-term survival in a large cohort of colorectal cancer patients from Germany. Eur J Epidemiol.

[16] Fedirko V, Riboli E, Tjønneland A (2012). Prediagnostic 25-hydroxyvitamin D, VDR and CASR polymorphisms, and survival in patients with colorectal cancer in western European ppulations. Cancer Epidemiol Biomarkers Prev.

[17] Väyrynen JP, Mutt SJ, Herzig KH (2016). Decreased preoperative serum 25-Hydroxyvitamin D levels in colorectal cancer are associated with systemic inflammation and serrated morphology. Sci Rep.

[18] Ng D, Tan R, Sultana R (2018). Prevalence of vitamin D deficiency in Chinese patients with early stage colorectal cancer. Ann Oncol.

[19] Reid D, Toole BJ, Knox S (2011). The relation between acute changes in the systemic inflammatory response and plasma 25-hydroxyvitamin D concentrations after elective knee arthroplasty. Am J Clin Nutr.

[20] Liberati A, Altman DG, Tetzlaff J (2009). The PRISMA statement for reporting systematic reviews and meta-analyses of studies that evaluate healthcare interventions: explanation and elaboration. BMJ.

[21] Wells GASB, O’Connell D, Peterson J, Welch V, Losos M, Tugwell P. The Newcastle-Ottawa Scale (NOS) for assessing the quality of nonrandomised studies in meta-analyses. https://www.ohri.ca/programs/clinical_epidemiology/oxford.asp. Accessed 25 Apr 2016.

[22] Applied statistics in. The pharmaceutical industry: with case studies using S-Plus. Springer Science & Business Media; 2013.

[23] Markotic A, Langer S, Kelava T (2019). Higher Post-operative Serum Vitamin D Level is Associated with Better Survival Outcome in Colorectal Cancer patients. Nutr Cancer.

[24] Wesselink E, Kok DE, de Wilt JHW (2021). Sufficient 25-Hydroxyvitamin D levels 2 years after Colorectal Cancer diagnosis are Associated with a lower risk of all-cause mortality. Cancer Epidemiol Biomarkers Prev.

[25] Vaughan-Shaw PG, Zgaga L, Ooi LY (2020). Low plasma vitamin D is associated with adverse colorectal cancer survival after surgical resection, independent of systemic inflammatory response. Gut.

[26] Zgaga L, Theodoratou E, Farrington SM (2014). Plasma vitamin D concentration influences survival outcome after a diagnosis of colorectal cancer. J Clin Oncol.

[27] Yang L, Chen H, Zhao M, Peng P (2017). Prognostic value of circulating vitamin D binding protein, total, free and bioavailable 25-hydroxy vitamin D in patients with colorectal cancer. Oncotarget.

[28] Akinci MB, Sendur MA, Aksoy S (2014). Serum 25-hydroxy vitamin D status is not related to osteopenia/osteoporosis risk in colorectal cancer survivors. Asian Pac J Cancer Prev.

[29] Mezawa H, Sugiura T, Watanabe M (2010). Serum vitamin D levels and survival of patients with colorectal cancer: post-hoc analysis of a prospective cohort study. BMC Cancer.

[30] Abrahamsson H, Meltzer S, Hagen VN (2021). Sex disparities in vitamin D status and the impact on systemic inflammation and survival in rectal cancer. BMC Cancer.

[31] Balci B, Kilinc G, Calik B, Aydin C (2021). The association between preoperative 25-OH vitamin D levels and postoperative complications in patients undergoing colorectal cancer surgery. BMC Surg.

[32] Bao Y, Li Y, Gong Y, Huang Q, Cai S, Peng J (2020). Vitamin D status and survival in stage II-III colorectal Cancer. Front Oncol.

[33] Wesselink E, Kok DE, Bours MJL (2020). Vitamin D, magnesium, calcium, and their interaction in relation to colorectal cancer recurrence and all-cause mortality. Am J Clin Nutr.

[34] Xingyu Chen. Correlation between preoperative vitamin D3 level and colorectal cancer. Fujian Medical University; 2020.

[35] Fedirko V, Mandle HB, Zhu W (2019). Vitamin D-Related genes, blood vitamin D levels and colorectal Cancer risk in western European populations. Nutrients.

[36] Meeker SM, Seamons A, Treuting PM (2020). Effect of chronic vitamin D Deficiency on the Development and Severity of DSS-Induced Colon cancer in Smad3-/- mice. Comp Med.

[37] Maalmi H, Ordóñez-Mena JM, Schöttker B, Brenner H (2014). Serum 25-hydroxyvitamin D levels and survival in colorectal and breast cancer patients: systematic review and meta-analysis of prospective cohort studies. Eur J Cancer.

[38] Toriola AT, Nguyen N, Scheitler-Ring K, Colditz GA (2014). Circulating 25-hydroxyvitamin D levels and prognosis among cancer patients: a systematic review. Cancer Epidemiol Biomarkers Prev.

[39] Gama R, Waldron JL, Ashby HL (2012). Hypovitaminosis D and disease: consequence rather than cause?. BMJ.

[40] Ovesen L, Andersen R, Jakobsen J (2003). Geographical differences in vitamin D status, with particular reference to European countries. Proc Nutr Soc.

[41] Liang Y, Jiang L, Chi X (2020). The association of serum vitamin D-binding protein and 25-hydroxyvitamin D in pre-operative and post-operative colorectal cancer. J Clin Lab Anal.

[42] Feldman D, Krishnan AV, Swami S, Giovannucci E, Feldman BJ (2014). The role of vitamin D in reducing cancer risk and progression. Nat Rev Cancer.

[43] Crescioli C, Minisola S, Vitamin D (2017). Autoimmunity and gender. Curr Med Chem.

[44] Patman G (2015). Colorectal cancer: male hormones increase the incidence of colonic adenomas. Nat Rev Gastroenterol Hepatol.

[45] Mousavi SE, Amini H, Heydarpour P, Amini Chermahini F, Godderis L (2019). Air pollution, environmental chemicals, and smoking may trigger vitamin D deficiency: evidence and potential mechanisms. Environ Int.

[46] Fernandes MR, Barreto WDR, Junior. Association between physical activity and vitamin D: A narrative literature review. Rev Assoc Med Bras (1992). 2017;63(6):550–556. 10.1590/1806-9282.63.06.550 28876433

[47] Vaughan-Shaw PG, Grimes G, Blackmur JP (2021). Oral vitamin D supplementation induces transcriptomic changes in rectal mucosa that are linked to anti-tumour effects. BMC Med.

[48] Mendes MM, Hart KH, Williams EL, Mendis J, Lanham-New SA, Botelho PB (2021). Vitamin D supplementation and sunlight exposure on serum vitamin D concentrations in 2 parallel, Double-Blind, randomized, placebo-controlled trials. J Nutr.

[49] Mentella MC, Scaldaferri F, Pizzoferrato M, Gasbarrini A, Miggiano GAD (2019). The Association of Disease Activity, BMI and Phase Angle with vitamin D Deficiency in patients with IBD. Nutrients.

[50] Saneei P, Salehi-Abargouei A, Esmaillzadeh A (2013). Serum 25-hydroxy vitamin D levels in relation to body mass index: a systematic review and meta-analysis. Obes Rev.

[51] Javed M, Althwanay A, Ahsan F (2020). Role of vitamin D in Colorectal Cancer: a holistic Approach and Review of the clinical utility. Cureus.

[52] Pandolfi F, Franza L, Mandolini C, Conti P (2017). Immune Modulation by vitamin D: special emphasis on its role in Prevention and Treatment of Cancer. Clin Ther.

[53] Schöttker B, Kuznia S, Laetsch DC (2020). Protocol of the VICTORIA study: personalized vitamin D supplementation for reducing or preventing fatigue and enhancing quality of life of patients with colorectal tumor - randomized intervention trial. BMC Cancer.

[54] Koole JL, Bours MJL, van Roekel EH (2020). Higher Serum Vitamin D Concentrations Are Longitudinally Associated with Better Global Quality of Life and less fatigue in Colorectal Cancer survivors up to 2 years after treatment. Cancer Epidemiol Biomarkers Prev.

[55] Kus T, Isbilen E, Aktas G, Arak H (2022). The predictive value of vitamin D follow-up and supplementation on recurrence in patients with colorectal cancer. Future Oncol.

[56] van Harten-Gerritsen AS, Balvers MG, Witkamp RF, Kampman E, van Duijnhoven FJ, Vitamin D (2015). Inflammation, and Colorectal Cancer Progression: a review of mechanistic studies and future directions for Epidemiological studies. Cancer Epidemiol Biomarkers Prev.

[57] Lasry A, Zinger A, Ben-Neriah Y (2016). Inflammatory networks underlying colorectal cancer. Nat Immunol.

[58] Seruga B, Zhang H, Bernstein LJ, Tannock IF (2008). Cytokines and their relationship to the symptoms and outcome of cancer. Nat Rev Cancer.

[59] Lopez-Candales A, Burgos PMH, Hernandez-Suarez DF, Harris D (2017). Linking chronic inflammation with cardiovascular disease: from normal aging to the metabolic syndrome. J Nat Sci.

[60] Bluethmann SM, Mariotto AB, Rowland JH (2016). Anticipating the silver tsunami: prevalence trajectories and Comorbidity Burden among Older Cancer survivors in the United States. Cancer Epidemiol Biomarkers Prev.

[61] Conway FJ, McMillan DC (2015). Plasma vitamin D concentration and survival in colorectal cancer: potential confounding by the systemic inflammatory response. J Clin Oncol.

[62] McMillan DC, Maguire D, Talwar D (2019). Relationship between nutritional status and the systemic inflammatory response: micronutrients. Proc Nutr Soc.

