# Supplementary material for: Plasma 25-hydroxyvitamin D deficiency in the peri-operative period is associated with survival outcome in colorectal cancer patients: a meta-analysis
Source: BMC Surg. 2024 Jun 12;24:180. doi: 10.1186/s12893-024-02473-5 (PMC11167935; doi:10.1186/s12893-024-02473-5)
Supplement: Supplementary file 1 — Supplementary Material 1 [file 12893_2024_2473_MOESM1_ESM.docx]

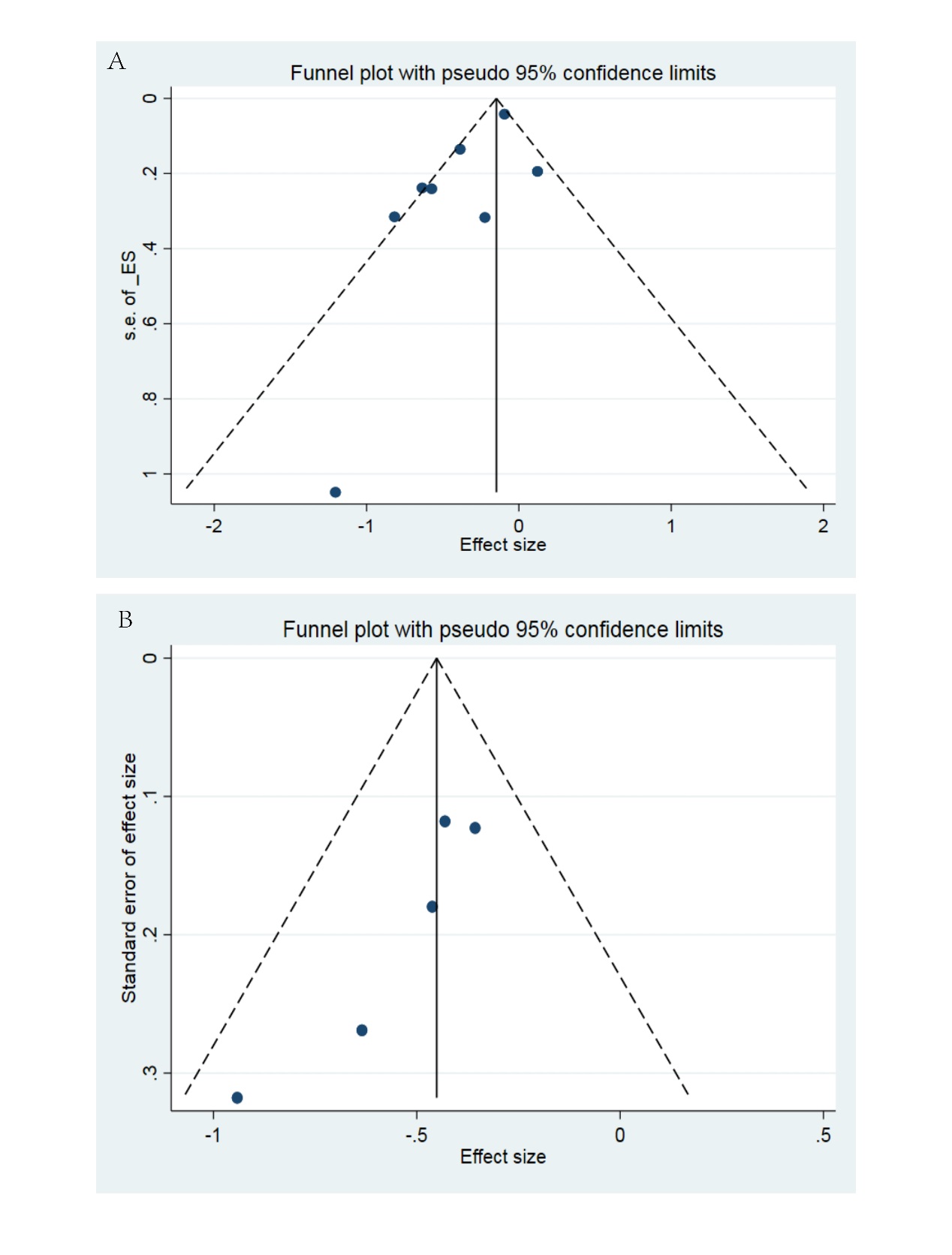


**Supplemental Fig. 1.** Funnel plots for publication bias in meta-analysis for prognostic outcome. **(A)** Funnel plots for publication bias in meta-analysis for overall survival. **(B)** Funnel plots for publication bias in meta-analysis for all-cause mortality.
